# AI-driven opportunistic screening for carotid atheroma calcification on dental radiographs

**DOI:** 10.1590/1807-3107bor-2026.vol40.088

**Published:** 2026-11-09

**Authors:** Camila Franzon CHINI, Igor Bispo de Moraes Coelho CORREIA, Matheus Virgílio da Silva FERREIRA, Bruno Scholles Soares DIAS, Nilce Santos de MELO, André Ferreira LEITE, Mylene Christine Queiroz de FARIAS

**Affiliations:** (a) Universidade de Brasília – UnB, School of Health Sciences, Department of Dentistry, Brasília, DF, Brazil; (b) Universidade de Brasília – UnB, Department of Electrical Engineering, Brasília, DF, Brazil.; (c) Texas State University, Department of Computer Science, San Marcos, TX, United States.

**Keywords:** Dentistry, Deep Learning, Diagnosis, Computer-Assisted, Vascular Calcification, Radiography, Panoramic

## Abstract

To develop a labeled image dataset of suspected carotid artery calcifications (CACs) on dental panoramic radiographs (PRs) and to propose a deep learning-based framework for their automated detection and segmentation. A retrospective dataset comprising 19,205 PRs obtained from our institution (between 2015 and 2023) was analyzed. After manual labeling, 372 images showing CACs and 322 controls were included. A hybrid architecture combining FasTVit, AttentionNet, and DC-UNet was implemented for classification, detection, and segmentation. Model performance was assessed using accuracy, precision, recall, the Dice coefficient, intersection over union (IoU), and area under the curve (AUC). The study evaluated two scenarios: one in which detection and segmentation were performed after classification, and another in which the classification was omitted. The classification model achieved an accuracy of 0.8780, a precision of 0.8779, a recall of 0.8795, and a specificity of, 0.8795. Incorporating the classification step improved precision from 0.48 to 0.93, accuracy from 0.79 to 0.97, and specificity from 0.75 to 0.98. The final framework achieved an area under the ROC curve of 0.96. The proposed hybrid artificial intelligence (AI) framework showed promising internal performance for the opportunistic detection and localization of radiographic findings suggestive of CACs on PRs. Within the limitations of this study, the proposed framework may serve as a complementary aid to dentists by flagging suspected CACs during routine interpretation of PRs and facilitating timely referral for confirmatory cardiovascular assessment.

## Introduction

Atherosclerosis is characterized by the accumulation of lipids, including cholesterol, within the arterial walls, leading to the formation of atheromas or atherosclerotic plaques. This process leads to arterial thickening and stiffening, impairing blood flow and potentially triggering myocardial infarctions and strokes.^1^ Evidence suggests that the risk of major adverse cardiovascular events is influenced not only by the presence of significant coronary blockages but also by the overall atherosclerotic plaque burden throughout the coronary arterial system.^2^ Atherosclerosis often goes unnoticed until it becomes severe because initial symptoms are typically absent and plaque accumulation occurs silently. As a result, atherosclerotic cardiovascular disease is the leading cause of mortality worldwide.^3^ Consequently, accurate and reliable diagnosis of atherosclerotic plaques prior to clinical manifestations is essential for the identification of high-risk patients.^4^

Within the context of routine healthcare, dental panoramic radiographs (PRs) are a rapid and low-cost imaging modality that requires minimal patient cooperation. PRs help identify not only dental health concerns but also systemic diseases.^5^ Calcification is a common component of atherosclerotic plaques. While calcification alone is not a definitive indicator of vascular disease, its presence suggests some degree of arteriosclerosis.^6^ In this context, carotid artery calcifications (CACs) can be detected on dental PRs, where they appear as irregular nodular radiopacities located inferior and posterior to the mandibular angle and the hyoid bone, adjacent to the cervical spine, typically at the level of the C3–C4 intervertebral space.^7,8^ As a result, this widely used and readily accessible imaging method represents a valuable tool for the opportunistic early detection and evaluation of CACs. Dentists can therefore play a vital role in the recognition and evaluation of these calcifications, thus contributing to reductions in morbidity and mortality rates.^9^

The combination of standard PR with artificial intelligence (AI) algorithms creates new opportunities for the diagnosis of systemic diseases.^9^ In particular, deep learning models can help identify potential CACs on PRs, which can be overlooked if the healthcare provider is not intentionally looking for them. AI algorithms can automate the analysis of dozens or even thousands of images, assisting professionals who might misclassify the presence of CACs due to the repetitive nature of image interpretation. As a result, AI algorithms can increase the probability of detecting CACs, thereby helping to prevent the progression of asymptomatic atherosclerotic disease in patients who may be unaware of their condition.^10^ In addition, the presence of CACs may help assess risk factors for atherosclerosis in other blood vessels, such as coronary and lower-extremity peripheral arteries.^9^ The aim of this study was to annotate an image dataset and develop an automated system for detecting CACs on PRs using a hybrid deep learning architecture.

## Methods

### Sample

A cross-sectional retrospective study was conducted using 19,205 digital PRs obtained from patients who attended our Department of Dental Radiology between 2015 and 2023. This study was carried out in accordance with the Declaration of Helsinki and was approved by the Research Ethics Committee (Approval no. 5.847.660; CAAE: 65772622.4.0000.0030*)*.

All PRs available in the institutional database (19,205 PRs) were screened by an experienced dentist (C.F.C.) according to the following inclusion and exclusion criteria.

The inclusion criteria were: a) individuals aged 18 years or older; b) high-quality PRs that allowed clear identification of anatomical landmarks, including the hyoid bone, the temporomandibular joint, cervical vertebrae, and the styloid process, allowing assessment of the region adjacent to the cervical spine at the C3–C4 intervertebral level, posterior and inferior to the mandibular angle, where CACs are commonly observed. The exclusion criteria were: a) patients with a documented history of trauma, disorders, syndromes, or anomalies in the region of interest (ROI); b) poor-quality PRs, including those with technical distortions, dimensional changes, or motion artifacts.

### Image acquisition

PRs were acquired using Kodak 8000C and the Ortophos (Sirona Dental Systems) units operating at 72-80 kVp and 15 mA, with an exposure time of approximately 13.9 seconds. The image dataset included two spatial resolutions: 308 images at 5,000×2,668 pixels (higher resolution) and 64 images at 2,855×1,350 pixels (lower resolution). The lower-resolution images were acquired earlier than the higher-resolution images and accounted for approximately 20% of the dataset.

### Image selection, annotation, and preprocessing

The images were assessed by a dentist experienced in the interpretation of CACs on PRs using a 29-inch LG ultra-wide full HD monitor under subdued lighting conditions. The region of interest for CAC evaluation was located inferior to the mandibular angle, adjacent to the C3-C4 intervertebral level (Figure 1). Manual annotation was performed by drawing bounding boxes using the Computer Vision Annotation Tool (CVAT).^11^

**Figure 1:**
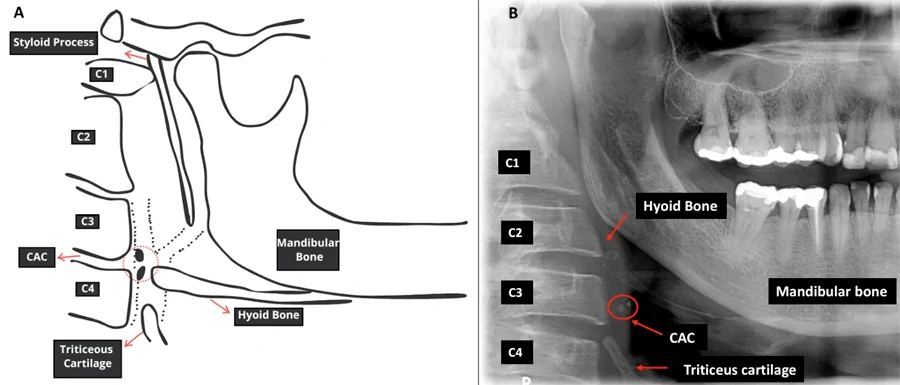
A - Schematic representation of the carotid artery region and its relationship to adjacent anatomical structures. Carotid artery calcification (CAC) is typically located at the carotid bifurcation, where the common carotid artery divides into the internal and external carotid arteries. B - Panoramic radiograph highlighting the corresponding anatomical structures.

All annotations were performed primarily by a single examiner trained in oral and maxillofacial radiology. In cases of uncertainty regarding the presence of suspected CACs, an oral and maxillofacial radiologist with more than 20 years of experience was consulted. However, no formal assessment of interobserver agreement was conducted, and the radiographic findings were not confirmed by complementary imaging methods, such as carotid doppler ultrasonography or computed tomography angiography.

During the selection and annotation processes, the experienced dentist applied established radiographic criteria for the differential diagnosis of CACs, including their characteristic vertical alignment with the cervical spine at the C3–C4 intervertebral space, irregular nodular morphology, and location posterior and inferior to the mandibular angle. Other soft-tissue calcifications that may occur in the cervical region were not annotated as separate classes in the dataset. Consequently, some degree of misclassification may have occurred, representing an inherent limitation of the study.

After screening and eligibility assessment, 372 PRs showing CACs and 322 PRs without CACs were included, resulting in a final dataset of 694 images. To facilitate the development of the AI model, CAC-negative images were selected to create a balanced dataset and reduce class imbalance during model training.

Preprocessing was performed with data augmentation, including random horizontal flips and resizing to 250×952. The images were normalized and equalized to standardize pixel intensity, followed by background removal using the rolling ball method,^12^ which enhances the contrast of CACs by removing low-intensity areas. The areas within the bounding boxes were processed using Otsu’s thresholding technique,^13^ generating initial binary masks in which CAC pixels were assigned a value of ‘1’ and all remaining pixels a value of ‘0’. To improve CAC detection, coarse segmentation masks were generated using a U-Net architecture,^14^ which was lightly trained on a limited set of images that had been reviewed and annotated by an expert.

### Dataset

A dataset was compiled comprising both demographic and clinical data, including sex, age, tooth count, smoking and alcohol use, weight, height, body mass index (BMI), lipid profile (high-density lipoprotein [HDL], and low-density lipoprotein [LDL]), in addition to chronic noncommunicable diseases, such as hypertension, osteoporosis, dyslipidemia, stroke, heart disease, diabetes, and nephropathy. Clinical data corresponding to each image were organized in a coded spreadsheet. Each image was assigned a unique numerical identifier, creating a structured and easily accessible dataset that linked the images to their corresponding medical records.

### Proposed framework

The proposed framework integrates three complementary deep learning models into a sequential pipeline for automated CAC detection on PRs. Figure 2 presents the block diagram of the proposed methodology. The pipeline comprises three main stages (classification, detection, and segmentation), followed by a merge step, as described next.

**Figure 2:**
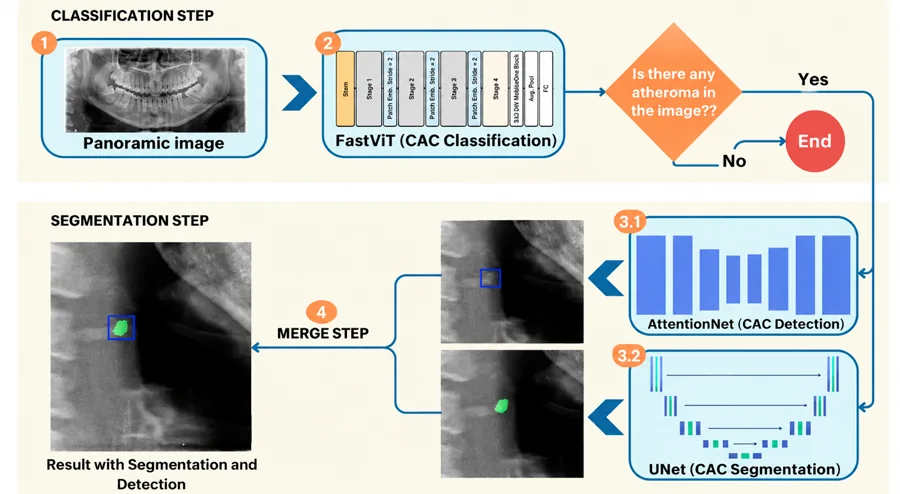
Overview of the automated CAC detection pipeline: (1) input panoramic radiographic, (2) CAC classification, (3.1) CAC localization, (3.2) CAC segmentation, and (4) merge step.

Classification: The first stage employs the FastVit architecture,^15^ which classifies each PR as CAC-positive or CAC-negative. This filtering step reduces the computational burden and minimizes false-positive detections in the subsequent stages. The initial model was initialized with ImageNet-pretrained weights ^16^ available in the Timm library^17^ (version 0.9.11) and adapted for binary classification of CAC-positive and CAC-negative PRs. The model was trained using the Adam optimizer with an initial learning rate of 0.001 and a StepLR scheduler (gamma = 0.7, step size = 1). Training was performed for 20 epochs with a batch size of 8 using a dataset comprising 257 CAC-negative and 297 CAC-positive PRs. The classification stage, illustrated in Box 2 at the top of Figure 2, is followed by two parallel and independent processing steps.

Detection: For PRs classified as CAC-positive, the AttentionNet architecture^18^ was used to localize the calcifications. This model generates bounding boxes around suspected CACs, consistent with radiologist annotations. For this task, we used the Faster R-CNN variant^19^ with a ResNet50 backbone,^20^ an attention mechanism, a feature pyramid network, using the hyperparameter settings described by the original authors.^18^ The input images were resized to 1333×800 pixels and augmented using random flipping. The training set comprised 297 images, while 75 images were assigned for validation. The model was trained for 100 epochs with a batch size of 2, following the configuration proposed by Zhu et al.^18^

Segmentation: To improve the detection of calcifications, we utilized the DC-UNet architecture to generate coarse segmentation masks.^21^ This approach involved semi-automatically generated annotation masks, which were further refined through expert review. DC-UNet offers enhancements over the traditional U-Net, featuring a CNN-based encoder-decoder design with residual blocks replacing conventional skip connections, along with the Tanimoto^21^ similarity metric, which complements the Jaccard index for more accurate grayscale image analysis. These enhancements improve the model’s ability to handle complex medical imaging scenarios, outperforming the segmentation effectiveness of classic U-Net. Because segmentation masks were unavailable, this stage was not evaluated separately. Instead, it was assessed only in conjunction with the detection stage as a way to improve detection accuracy.

To clarify the dataset organization across the different stages of the pipeline, model-specific partitions were created according to the type of annotation available for each task. The classification model (FastViT) was trained and tested using the complete dataset of 694 PRs, with 554 images assigned to training and 140 images reserved for testing. For the detection stage (AttentionNet/Faster R-CNN), only CAC-positive PRs (n = 372) were included in the training procedure, as bounding-box annotations were available exclusively for these images. This subset was divided into a training set of 297 images and a validation set of 75 images. The segmentation-refinement stage (DC-UNet) also used the CAC-positive subset, as pixel-level masks were generated from the annotated lesion regions. No independent test set was used to evaluate the segmentation module separately. Therefore, its role should be regarded as a refinement component within the integrated pipeline. All partitions were performed at the patient level to prevent overlap between training, validation, and test subsets (Figure 2).

Merge step: The final prediction was generated by merging the outputs of AttentionNet and DC-UNet. Diagnostic accuracy was enhanced by retaining only the segmented areas that fall within the identified bounding boxes.

All models were implemented in PyTorch and trained on a workstation equipped with an Intel E5-2620 v4 CPU and an NVIDIA Quadro P6000 GPU. The performance of each stage (classification and detection) was assessed separately and within the integrated framework.

## Results

Among the 19,205 PRs screened from the institutional database, 372 showed radiographic findings suggestive of CACs, representing an overall prevalence of 1.93% in the source population (372/19,205 = 0.01936). For model development, 372 CAC-positive PRs and 322 CAC-negative controls were included, resulting in a final analytical dataset of 694 images. The positive cases were manually annotated using CVAT, yielding 574 labeled CAC regions, as more than one calcification could be present on a single radiograph. Within the positive group, 128 cases exhibited left-sided calcifications, 133 showed right-sided calcifications, and 111 exhibited bilateral calcifications. The CAC-positive group included 229 women (61.5%) and 143 men (38.4%), with a mean age of 58.6 years (range: 23–90 years; SD = 13.3).

The FastViT-based model demonstrated robust performance in distinguishing CAC-positive from CAC-negative cases. As shown in Table 1, the model achieved an accuracy of 0.878, a precision of 0.878, a recall (sensitivity) of 0.880, an F1-score of 0.878, and a specificity of 0.880. These balanced values highlight the reliability of the model in detecting the presence of CACs. The confusion matrix shown in Figure 3 further illustrates the classification performance: the model correctly identified 58 CAC-negative cases and 65 CAC-positive cases, with only 10 false negatives and seven false positives.

**Table 1:** Performance metrics of the FastViT-based model during the classification stage.

Metric	Value
Accuracy	0.8780
Precision	0.8779
Recall/Sensitivity	0.8795
F1-Score	0.8783
Specificity	0.8795

**Figure 3:**
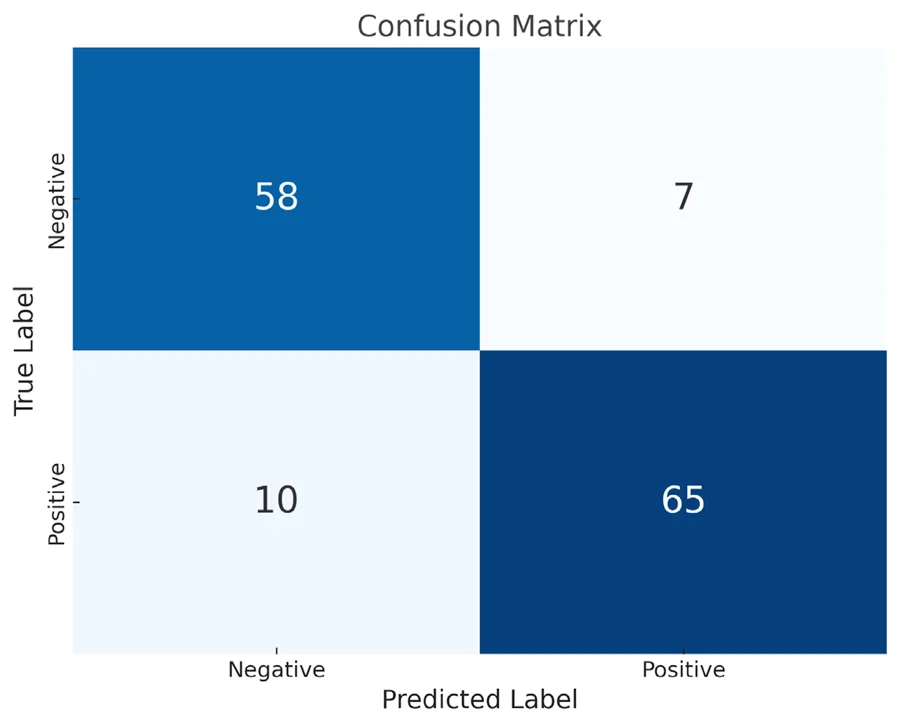
Confusion matrix showing the performance of the FastViT-based model in classifying panoramic radiographs as CAC-positive or CAC-negative.

The AttentionNet model (Box 3.1 in Figure 2) was evaluated for its ability to localize CACs by generating bounding boxes around the calcifications. Unlike classification tasks, detection performance cannot be measured solely using metrics such as accuracy, precision, or recall, as it requires comparison of the predicted bounding boxes with expert annotations. Therefore, standard object detection metrics, average precision (AP) and average recall (AR), were used at different intersection over union (IoU) thresholds.

The IoU was calculated as the overlap between the predicted and ground-truth bounding boxes, divided by the area of their union, with values closer to 1 indicating greater localization accuracy.^22^ A prediction was considered correct when its IoU exceeded the specified threshold. Following the COCO standard,^22^ bounding boxes were categorized as small (< 32 pixels^2^), medium-sized (32–96 pixels^2^), or large (> 96 pixels^2^). As presented in Table 2, the model achieved an AP of 0.414 and an AR of 0.559 for medium-sized CACs at IoU thresholds ranging from 0.50 to 0.95. These findings indicate that the model was effective in detecting medium-sized calcifications, which accounted for the majority of cases in the dataset.

**Table 2:** Validation metrics of the AttentionNet model during the detection stage.

Metric	IoU	Area	Value
AP	0.50:0.95	All	0.321
AP	0.50	All	0.638
AP	0.75	All	0.206
AP	0.50:0.95	Medium	0.414
AP	0.50:0.95	Large	0.264
AR	0.50:0.95	All	0.475
AR	0.50:0.95	Medium	0.559
AR	0.50:0.95	Large	0.413

As previously noted, no quantitative results are presented for the segmentation stage (Box 3.2 in Figure 2) because ground-truth calcification masks were unavailable and the dataset was too small to support statistically robust model training and evaluation. Consequently, our analysis was limited to overall performance, including separate evaluation of each model and evaluation of the integrated framework. Representative qualitative results are shown on the left side of Figure 4, where the detected bounding boxes (in green) and the corresponding segmentation masks (in blue) illustrate the output of the segmentation stage.

**Figure 4:**
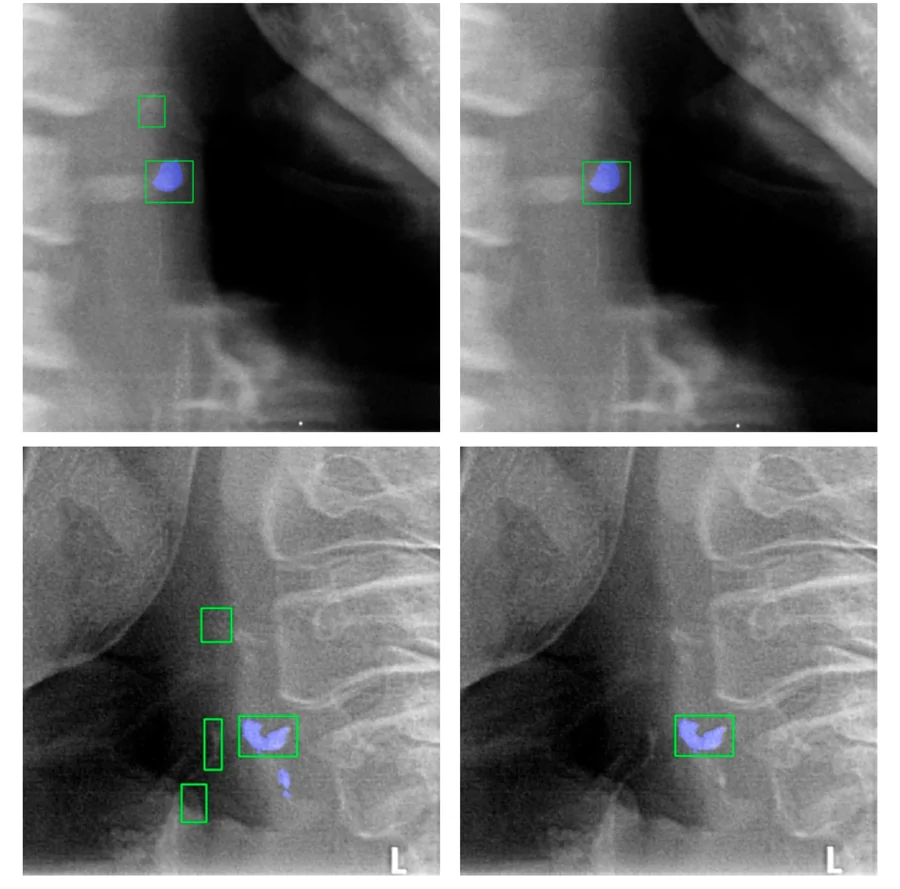
Comparison of CAC detection before (left) and after (right) the merge step.

Two experimental configurations were evaluated: a) detection and segmentation only, and b) an initial classification stage followed by detection and segmentation. As shown in Table 3, inclusion of the classification stage substantially improved precision (from 0.48 to 0.93), accuracy (from 0.79 to 0.97), and specificity (from 0.75 to 0.98), whereas recall decreased slightly (from 0.97 to 0.93). The images on the right side of Figure 4 illustrate the ability of the proposed framework to accurately localize and delineate CACs, demonstrating both successful detections and a marked reduction in false-positive predictions following the addition of the classification stage.

**Table 3:** Performance comparison with and without the classification stage.

Metric	Without classification	With classification
Precision	0.48	0.93
Recall	0.97	0.93
Accuracy	0.79	0.97
Specificity	0.75	0.98

## Discussion

This study demonstrates the feasibility of a hybrid deep learning framework for the detection of CACs on PRs. To the best of our knowledge, it is the first hybrid multistage methodology specifically designed for CAC detection on PRs, integrating complementary image processing techniques with deep learning-based modules to improve diagnostic accuracy and robustness. Collectively, these advances establish a new benchmark for artificial intelligence-driven opportunistic cardiovascular risk assessment in dental imaging.

A crucial element contributing to our findings is the development of a dataset specifically designed for CAC detection, comprising 19,205 PRs, of which 372 PRs were CAC-positive. More specifically, although previous research has utilized deep learning in PR datasets, ranging from smaller samples of 34,^23^ 65,^24^ 179^9^ to 20,000–23,000 images, these efforts focused on the detection of various dental anomalies and systemic conditions.^10,25^ Only Yılmaz^26^ used a substantially larger dataset of 30,883 PRs to compare different segmentation models, but their study did not specifically target CAC detection.

In the present study, the relatively large dataset specifically developed for CAC detection strengthens the reliability of the findings and reduces the potential for sampling bias. Previous studies have adopted a variety of approaches for CAC detection on PRs. The first attempt applied a conventional top-hat filter to a small dataset of 34 PRs, using the mandibular curvature as a reference point, and achieved a sensitivity of 93.6%.^23^ Using a limited dataset of 65 PRs, Kats et al.^24^ developed a deep learning model that achieved a sensitivity of 75%, a specificity of 80%, and a precision of 83%. Song et al.^10^ analyzed 352 images containing various soft tissue calcifications (including CACs, lymph node calcifications, and sialoliths). Using AI alone, they reported a sensitivity of 77.4%, a specificity of 71.7%, and a precision of 72.7%. When combined with radiologist assessments, performance improved substantially, reaching a sensitivity of 96.9%, a specificity of 81.6%, and an accuracy of 84.1%.

Lee et al.^25^ evaluated multiple abnormalities and, for CAC detection, reported an accuracy of 42.3%, a sensitivity of 95.8%, and a specificity of 95.9%. Their dataset included 72 labeled positive cases, 126 predicted cases, 69 true positives, 1,328 negative cases, and 1,274 true negatives. Amitay et al.^9^ tested CNN architectures such as DenseNet169, InceptionResNetV2, and Efficient NetV2M on 179 PRs, achieving a precision of 84%, a sensitivity of 82%, and a specificity of 97%. Most recently, Yoo et al.^27^ proposed a cascaded deep learning framework applied to 400 PRs. CAC annotations were performed by radiologists using CT scans as the reference standard. Their approach achieved an AUC of 99.6%, a precision of 98.5%, a sensitivity of 98%, and a specificity of 98.8%.

The present study advances the field by introducing an enhanced deep learning framework that integrates attention mechanisms into a U-Net-based architecture to specifically improve the accuracy of CAC detection on PRs. In contrast to previous studies that predominantly relied on a single AI model, the proposed framework employed a novel two-stage detection algorithm, substantially enhancing the precision and robustness of CAC detection on PRs. Moreover, the framework provided a more comprehensive and fully automated solution that does not depend on additional imaging modalities or complementary examinations, thereby rendering it particularly suitable for the early detection of systemic conditions in clinical practice. As an additional contribution, we make both the source code and the dataset publicly available through an anonymous repository.

Annotation strategies have varied in previous studies. Kats et al.^24^ and Song et al.^10^ reported manual labeling of calcifications but did not specify the tools used. Similarly, Lee et al.^25^ and Amitay et al.^9^ indicated that images were annotated by oral radiologists but did not describe the annotation platform. In contrast, Sawagashira et al.^23^ used the Canny edge detection algorithm, while Yoo et al.^27^ utilized the Supervisely platform for classification and segmentation. These variations underscore the lack of standardization in annotation methodologies, which can affect dataset quality and model performance. In the present study, all images were manually annotated by an experienced dentist using bounding boxes and refined pixel-wise segmentation masks via the CVAT tool.^11^ This rigorous approach, combined with expert validation, enhanced the accuracy and reliability of the ground-truth dataset, representing a methodological advantage over previous studies and supporting robust training and evaluation of deep learning models.

A key consideration regarding the clinical applicability of the proposed model is the potential for misclassification of other soft-tissue calcifications in the cervical region, particularly triticeous cartilage and superior thyroid horn calcifications. Although the examiner used strict anatomical and morphological criteria to distinguish suspected CACs from other cervical calcifications during image annotation, the model may occasionally misclassify these structures. This limitation should be considered in light of the intended use of the framework as an opportunistic screening tool rather than a definitive diagnostic system. Actually, previous studies have also used PRs as an initial imaging modality for identifying radiographic findings suggestive of CACs, based mainly on anatomical location and radiographic morphology. Therefore, model-positive findings should be interpreted as suspicious radiographic findings that warrant professional review, with referral for confirmatory medical evaluation when clinically appropriate.

The high specificity observed in the present study should be interpreted with caution, as other cervical soft-tissue calcifications and calcified anatomical structures were not annotated as separate classes. Therefore, the present findings do not demonstrate the model’s robustness against all radiographic mimickers of CAC. Nevertheless, the proposed framework is intended for opportunistic screening rather than for the definitive diagnosis of carotid atherosclerosis. From this perspective, the model may serve as a decision-support tool for identifying radiographic findings suggestive of CACs that warrant professional review, with referral for medical evaluation or confirmatory imaging when appropriate.

Hybrid architectures have demonstrated superior performance in medical imaging tasks.^28^ In this study, we developed a hybrid framework that integrates FastViT,^15^ AttentionNet,^18^ and DC-Unet.^14^ To our knowledge, this is the first study to apply a hybrid approach for CAC detection on PRs, representing methodological advances and an innovative tool for both clinical and research applications. Moreover, the outputs of the detection and segmentation models were combined through a merge step, retaining only the regions segmented within the detected bounding boxes. This two-stage process effectively reduced false positives while enhancing the precision and overall CAC detection performance.

The classification stage based on the FastViT architecture^15^ demonstrated robust performance, with all evaluation metrics approaching 0.88. These findings indicate the model’s ability to reliably distinguish CAC-positive from CAC-negative PRs, thus reducing the risk of misclassification. Compared with earlier studies that used conventional CNN architectures for CAC identification, such as those by Kats et al.^24^ and Amitay et al.,^9^ the present model achieved competitive or superior results while offering greater computational efficiency. These findings highlight the potential of the classifier as an effective first stage in automated CAC detection pipelines, filtering images before subsequent detection and segmentation tasks. To the best of our knowledge, this is the first application of a Fast-ViT-based architecture for CAC detection on PRs, extending the use of transformer-based models to dentomaxillofacial image analysis.

Most previous studies in dentomaxillofacial radiology have relied on convolutional neural networks (CNNs), such as U-Net, ResNet, and EfficientNet, for lesion detection and segmentation. These architectures, however, have intrinsic limitations in modeling long-range spatial dependencies, which are particularly relevant in panoramic radiography. The Fast-ViT model, employed in this study builds upon the Vision Transformer family by combining convolutional embeddings with efficient attention mechanisms. The ability of this model to capture global contextual relationships while maintaining computational efficiency represents a methodological advance that extends the application of state-of-the-art computer vision techniques to dental radiology research.

The AttentionNet model^18^ effectively localized CACs on PRs using bounding boxes, and achieved the best performance for medium-sized calcifications, which are clinically significant as they accounted for the majority of cases in the dataset. According to Garay et al.,^29^ the key diagnostic criteria for CACs include anatomical location, distribution, and the number, size, and shape of calcifications. Consequently, the present study evaluated the detection of CACs in different lesion sizes, allowing a more detailed assessment of model performance according to calcification dimensions. The stratified analysis conducted in the present study provided clinically meaningful insights, as the radiographic appearance of CACs can vary substantially depending on lesion extent. Among previous studies, only that conducted by Yoo et al.^27^ applied a similar size-based evaluation, whereas most reported overall findings without stratification. By using such approach, the present study offered a more comprehensive assessment of the applicability of the framework in various clinical scenarios.

Although detection performance was lower for small and large calcifications, the model consistently approximated the anatomical location of CACs, with bounding boxes generally aligned with expert annotations. In object detection, a true positive requires a minimum degree of overlap between the predicted bounding box and the ground-truth annotation, with values closer to 1 indicating more accurate localization.^30,31^ Although the bounding boxes generated by the model did not always perfectly align with expert annotations, the system consistently identified suspicious regions. From a clinical perspective, this is highly relevant, as it directs the dentist’s attention to specific areas of the PR that might otherwise be overlooked, thus facilitating earlier recognition and referral for further evaluation.

The segmentation stage provided complementary information by delineating calcification boundaries and enabling lesion detection and quantification. The DC-UNet algorithm^21^ (Box 3.2 in Figure 2) performs pixel-based image segmentation, a process in which the image pixels that belong to a calcification are marked as ‘true’ (one) in the output mask and pixels without calcification are marked as ‘false’ (zero). This architecture is adapted from the widely used Unet, renowned for its effectiveness in biomedical image segmentation,^14^ and its variant, DC-Unet,^21^ incorporates dual-channel blocks to overcome certain limitations of the standard UNet, enhancing calcification delineation while maintaining computational efficiency.

The integration of FastViT, AttentionNet, and DC-Unet into the proposed framework improved overall pipeline performance compared with the detection/segmentation configuration alone. The inclusion of the classification stage enhanced precision (from 0.48 to 0.93), accuracy (from 0.79 to 0.97), and specificity (from 0.75 to 0.98), with a modest reduction in recall (from 0.97 to 0.93). This improvement, however, should be interpreted with caution. In a sequential cascade design, the initial classifier removed a substantial proportion of CAC-negative images before the detection stage, thereby reducing false-positive predictions and improving precision, accuracy, and specificity. Therefore, the observed performance gains should not be attributed solely to improved lesion detection. A more conservative interpretation is that the cascade improved the system’s practical utility by reducing unnecessary detection runs and limiting false-positive prompts for the reviewing professional, at the cost of a small reduction in recall. In the context of opportunistic screening, the clinical acceptability of this trade-off should be evaluated according to the intended use of the tool and confirmed through prospective external validation studies. Future studies should also assess the behavior of the cascade under different prevalence scenarios using simulation analyses and independent datasets.

Finally, Figure 5 presents the precision-recall and receiver operating characteristic curves. The precision-recall curve demonstrates the model’s ability to maintain high precision at different recall levels, indicating its effectiveness in accurately detecting CACs while minimizing false positives. Nevertheless, precision declined as recall increased because of misclassifications. On the other hand, the receiver operating characteristic curve yielded an AUC of 0.96, indicating excellent overall performance, reflecting the model’s strong capability to differentiate between CAC-positive and CAC-negative PRs, with performance well above chance level.

**Figure 5:**
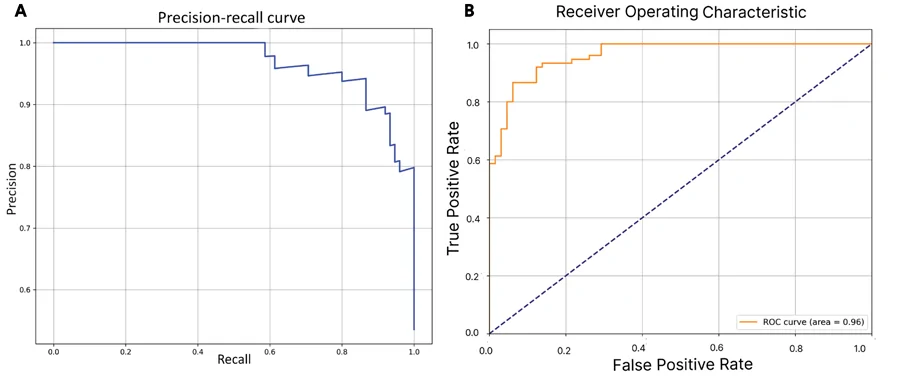
Performance evaluation of the classification model. (a) Precision-recall curve. (b) Receiver operating characteristic (ROC) curve.

A limitation of the present study is the absence of formal external validation using an independent dataset. Although the model was developed using PRs acquired from two different imaging systems, which may have introduced some variability in image characteristics, all data were obtained from a single institution. Therefore, the findings should be interpreted with caution. Further studies involving independent and multicenter datasets are needed to confirm the generalizability and robustness of the proposed approach across different populations, imaging devices, and acquisition protocols. In addition, we are going to conduct a prospective study to assess the clinical applicability of the proposed AI system and its potential to assist dental professionals in identifying radiographic findings suggestive of CACs during their routine practice.

Consistent with previous studies,^9,24,25,27,28,32^ suspected CACs were identified on PRs based on their radiographic appearance and anatomical location, without confirmation by carotid ultrasonography or computed tomography angiography. Therefore, the reference standard adopted in the present study should be interpreted as an expert radiographic reference rather than a definitive vascular diagnosis. This represents an important limitation, as the two-dimensional nature of panoramic radiography, anatomical superimposition, image distortion, and the presence of other cervical soft-tissue calcifications or calcified anatomical structures may lead to occasional misclassification.

Nevertheless, panoramic radiography is a widely available, low-cost imaging modality routinely used in dental practice that may allow the incidental detection of radiographic findings suggestive of CACs without additional radiation exposure or cost. In this context, the objective of the present study was not to establish a definitive diagnosis of carotid atherosclerosis, but rather to explore the potential of panoramic radiographs as a tool for opportunistic screening. Similar to previous studies, suspected CACs were identified based on established radiographic criteria, including their location near the C3-C4 intervertebral level, posterior and inferior to the mandibular angle, and their irregular nodular radiopaque appearance. Thus, the proposed framework should be understood as a decision-support tool intended to flag suspicious radiographic findings for professional review, with referral for medical evaluation or confirmatory imaging when clinically appropriate.

Future studies should prioritize external validation of the proposed framework using independent and heterogeneous datasets to confirm its generalizability and clinical applicability. Multicenter collaborations and prospective cohort studies will be essential to overcome limitations and ensure robust model performance across diverse imaging conditions and populations. Clinical implementation of the proposed framework will require: (i) prospective external validation in multicenter cohorts; (ii) explicit modeling of other soft-tissue calcifications; (iii) paired confirmatory imaging, such as doppler ultrasonography or CT angiography, in a validation subsample; and (iv) prospective evaluation of the impact of model-assisted reading on dentists’ recognition and referral behavior. Until these conditions are met, the proposed framework should be regarded as a research-stage decision-support prototype rather than a deployable diagnostic instrument. In summary, the proposed framework provides an automated and reproducible approach for the opportunistic identification of radiographic findings suggestive of CACs on PRs, with the potential to assist radiologists and dentists during routine image interpretation.

## Conclusion

This study demonstrated the feasibility of a hybrid deep learning framework for the opportunistic identification of radiographic findings suggestive of CAC on PRs. The proposed pipeline combined image-level classification, localization, and segmentation-based refinement, achieving promising internal performance while reducing false-positive detections after the inclusion of the classification stage. The development of a curated and expert-annotated image dataset also provides a valuable resource for future research in dental and maxillofacial radiology.

Nevertheless, the proposed framework should be regarded as a methodological proof of concept rather than a clinically deployable diagnostic tool. Its performance was evaluated using a single-center retrospective dataset, without external validation or confirmatory vascular imaging. Therefore, multicenter prospective studies with multi-class annotation of cervical soft-tissue calcifications and paired confirmatory imaging are needed to establish the robustness, generalizability, and clinical utility of the framework before routine clinical implementation.

The framework integrates three distinct machine learning models that first detect CACs and then perform lesion segmentation. The proposed AI model showed promising potential for opportunistic screening of carotid artery calcifications on PRs; however, external validation studies are needed to further establish its robustness and generalizability.

## Data Availability

The datasets generated during and/or analyzed during the current study are available from the corresponding author on reasonable request.

## References

[1] Zhao L, Ma D, Wang L, Su X, Feng L, Zhu L (2023). Metabolic changes with the occurrence of atherosclerotic plaques and the effects of statins. Front Immunol.

[2] Stone PH, Libby P, Boden WE (2023). Fundamental pathobiology of coronary atherosclerosis and clinical implications for chronic ischemic heart disease management-the plaque hypothesis: a narrative review. JAMA Cardiol.

[3] Libby P (2021). The changing landscape of atherosclerosis. Nature.

[4] Mushenkova NV, Summerhill VI, Zhang D, Romanenko EB, Grechko AV, Orekhov AN (2020). Current advances in the diagnostic imaging of atherosclerosis: insights into the pathophysiology of vulnerable plaque. Int J Mol Sci.

[5] Almeida FT, Gianoni-Capenakas S, Rabie H, Figueiredo R, Pacheco-Pereira C (2024). The use of panoramic radiographs to address the oral health needs of vulnerable Canadian populations. Can J Dent Hyg.

[6] Almog DM, Horev T, Illig KA, Green RM, Carter LC (2002). Correlating carotid artery stenosis detected by panoramic radiography with clinically relevant carotid artery stenosis determined by duplex ultrasound. Oral Surg Oral Med Oral Pathol Oral Radiol Endod.

[7] Friedlander AH, Lande A (1981). Panoramic radiographic identification of carotid arterial plaques. Oral Surg Oral Med Oral Pathol.

[8] Nasseh I, Aoun G (2018). Carotid artery calcification: a digital panoramic-based study. Diseases.

[9] Amitay M, Barnett-Itzhaki Z, Sudri S, Drori C, Wase T, Abu-El-Naaj I (2023). Deep convolution neural network for screening carotid calcification in dental panoramic radiographs. PLOS Digit Health.

[10] Song YB, Jeong HG, Kim C, Kim D, Kim J, Kim HJ (2022). Comparison of detection performance of soft tissue calcifications using artificial intelligence in panoramic radiography. Sci Rep.

[11] Corporation (2022). Computer Vision Annotation Tool. cvat-ai/.

[12] Sternberg (1983). Biomedical image processing. Computer.

[13] Sezgin M, Sankur B (2004). Survey over image thresholding techniques and quantitative performance evaluation. J Electron Imaging.

[14] Ronneberger O, Fischer P, Brox T, Navab N, Hornegger J, Wells WM, Frangi AF (2015). MICCAI 2015.

[15] Vasu PK, Gabriel J, Zhu J, Tuzel O, Ranjan A (2023). FastViT: a fast hybrid vision transformer using structural reparameterization. arXiv.

[16] Deng J, Dong W, Socher R, Li LJ, Li K, Fei-Fei L (2009). ImageNet: a large-scale hierarchical image database.

[17] Wightman R, Raw N, Soare A, Arora A, Ha C, Reich C (2025). Rwightman/pytorch image models.

[18] Zhu X, Cheng D, Zhang Z, Lin S, Dai J (2019). An Empirical Study of Spatial Attention Mechanisms in Deep Networks. arXiv.

[19] Ren S, He K, Girshick R, Sun J (2015). Faster R-CNN: towards real-time object detection with region proposal networks. arXiv.org.

[20] He K, Zhang X, Ren S, Sun J (2016). Deep residual learning for image recognition.

[21] Lou A, Guan S, Loew M (2020). DC-UNet: rethinking the u-net architecture with dual channel efficient CNN for medical images segmentation.

[22] COCO - Common Objects in Context https://cocodataset.org/#home.

[23] Sawagashira T, Hayashi T, Hara T, Katsumata A, Muramatsu C, Zhou X (2011). An automatic detection method for carotid artery calcifications using top-hat filter on dental panoramic radiographs. Annu Int Conf IEEE Eng Med Biol Soc.

[24] Kats L, Vered M, Zlotogorski-Hurvitz A, Harpaz I (2019). Atherosclerotic carotid plaque on panoramic r adiographs: neural network detection. Int J Comput Dent.

[25] Lee S, Kim D, Jeong HG (2022). Detecting 17 fine-grained dental anomalies from panoramic dental radiography using artificial intelligence. Sci Rep.

[26] Yilmaz BG, Altun S, Bayrakdar IS (2026). Evaluation of deep learning-based segmentation models for carotid artery calcification detection in panoramic radiographs. Oral Radiol.

[27] Yoo SW, Yang S, Kim JE, Huh KH, Lee SS, Heo MS (2024). CACSNet for automatic robust classification and segmentation of carotid artery calcification on panoramic radiographs using a cascaded deep learning network. Sci Rep.

[28] Arzani S, Soltani P, Karimi A, Yazdi M, Ayoub A, Khurshid Z (2025). Detection of carotid artery calcifications using artificial intelligence in dental radiographs: a systematic review and meta-analysis. BMC Med Imaging.

[29] Garay I, Netto HD, Olate S (2014). Soft tissue calcified in mandibular angle area observed by means of panoramic radiography. Int J Clin Exp Med.

[30] Zong Z, Song G, Liu Y (2023). DETRs with collaborative hybrid assignments training.

[31] Fang Y, Wang W, Xie B, Sun Q, Wu Km, Wang X (2022). EVA: Exploring the Limits of masked visual representation learning at scale. arXiv.

[32] Kuwada C, Mitsuya Y, Fukuda M, Yang S, Kise Y, Mori M (2026). Area detection improves the person-based performance of a deep learning system for classifying the presence of carotid artery calcifications on panoramic radiographs. Oral Radiol.

[33] Sutter W, Berger S, Meier M, Kropp A, Kielbassa AM, Turhani D (2018). Cross-sectional study on the prevalence of carotid artery calcifications, tonsilloliths, calcified submandibular lymph nodes, sialoliths of the submandibular gland, and idiopathic osteosclerosis using digital panoramic radiography in a Lower Austrian subpopulation. Quintessence Int.

[34] Schneider B, Grün P, Degel U, Ströbele D, Bandura P, Pfaffeneder-Mantai F (2024). Influence of clinical experience in detecting calcifications of the head and neck region on panoramic radiographs: an app-based evaluation. Ann Med Surg (Lond).

